# An Improved Method of Minimizing Tool Vibration during Boring Holes in Large-Size Structures

**DOI:** 10.3390/ma14164491

**Published:** 2021-08-10

**Authors:** Krzysztof J. Kaliński, Marek A. Galewski, Michał R. Mazur, Natalia Stawicka-Morawska

**Affiliations:** Faculty of Mechanical Engineering and Ship Technology, Gdansk University of Technology, G. Narutowicza 11/12, 80-233 Gdansk, Poland; krzysztof.kalinski@pg.edu.pl (K.J.K.); michal.mazur@pg.edu.pl (M.R.M.); natalia.morawska@pg.edu.pl (N.S.-M.)

**Keywords:** boring, vibration suppression, simulations, the best spindle speed, experimental identification

## Abstract

The paper presents a thoroughly modified method of solving the problem of vibration suppression when boring large-diameter holes in large-size workpieces. A new approach of adjusting the rotational speed of a boring tool is proposed which concerns the selection of the spindle speed in accordance with the results of the simulation of the cutting process. This streamlined method focuses on phenomenological aspects and involves the identification of a Finite Element Model (FEM) of a rotating boring tool only and validating it with a real object, while dispensing with discrete modelling of a completely rigid workpiece. In addition, vibrations in the boring process in all directions were observed, which implies a geometric nonlinearity of the process model. During the simulation, the values of the Root Mean Square (RMS) of the time plots and the dominant values of the “peaks” in the displacement amplitude spectra were obtained. The effectiveness of the method was demonstrated using a selected mechatronic design technique called Experiment-Aided Virtual Prototyping (E-AVP). It was successfully verified by measuring the roughness of the indicated zone of the workpiece surface. The economic profitability of implementing the method in the production practice of enterprises dealing with mechanical processing is also demonstrated.

## 1. Introduction

The relative vibrations between the tool and the workpiece are recognized as the main cause of various problems detected during large-size structures machining [1]. Under certain circumstances occurring in the boring process, they can lead to a loss of system stability and the appearance of self-excited chatter vibrations, due to process and structure dynamic interactions resulting in modulated chip thickness [2]. These can cause a reduction in the overall machine tool performance or the quality of the workpiece surface, increased tool wear, especially when boring large diameter holes [3]. In some extreme cases it can even lead to the destruction of the tool or workpiece. To increase the milling efficiency while maintaining the surface quality and minimizing the vibration level, parameters of the machining process should be adjusted [4].

There are many scientific studies devoted to the problems of vibration reduction in the boring process [5]. For example, chatter is a limiting factor during boring of deep holes with long slender boring bars and may be effectively damped by the magnetorheological damper at different machining conditions for boring of Inconel 718 and Al 7075 workpieces, but the method was presented only for a case of small boring tool (length 300 mm, diameter 25 mm) [6]. In [7] Miguélez et al. presented the behavior of boring bars, which were modelled as cantilever Euler–Bernoulli beams, with a passive dynamic vibration absorber for chatter suppression. Only the first mode of vibration of the bars was considered. Bansal and Law presented (in [8]) a receptance coupling-based method to optimally tune and place a tuned mass damper on a similar slender boring bar. Chatter-free depth of cut for boring was increased a few times, which is a significant improvement. The considerations concerned a rotating workpiece, the spindle speed of which has not been identified. In [9] Yadav et al. described a receptance coupling approach in which the substructural receptances of the boring bar modelled as a cantilevered Euler–Bernoulli beam are combined with the substructural receptances of a damper modelled as a rigid mass integrated anywhere within the bar. The improvement of the damping capability of boring tools by using impact dampers was investigated in [10]. Chatter vibration elimination during deep hole boring using slender bars was considered in [11,12]. Composite material may have much higher stiffness and damping in comparison to metal, and tests of such properties for chatter suppression were investigated in [13,14,15,16]. The design and testing of a new dynamic system for boring, based on a tuned holder that increases the dynamic stiffness of slender boring bars by matching the holder natural frequency to the clamped-free boring bar natural frequency, reduced susceptibility to chatter [17]. However, the considerations made there were not verified in the real machining process. In [18] design of the low-cost damped tool holder using different types of high-density materials such as copper and brass to suppress the chatter of boring operations is investigated. The design of an anisotropic boring tool as a chatter suppression method was presented in [19,20]. The obtained theoretical and experimental results showed that using piezoelectric shunt damping it is possible to significantly increase the stability margin in boring operations during tests limited to small boring bars and rotating parts with undefined spindle speed [2]. An adaptive sliding mode control approach is presented to suppress the chatter in the boring process in the presence of uncertainties of model and dynamics [21]. The industrialized version of the boring bar with embedded sensors gives valuable insight into the cutting process during which chatter and excessive insert wear can be detected [22]. The vibration measurement gives an indication of the quality of the machined surface and thus shows the potential of the technology. The results of machining in both Maraging 250 and steel at a cutting speed of 120 m/min, a hole diameter of about 90 mm and a slender bar, whose length is unidentified, are presented. In order to meet the requirements of boring holes about 22 mm diameter with rotating bars, overhang lengths from 160 mm to 420 mm, at high rotational speed (2500 rpm), a boring bar was designed and manufactured with high stiffness pitch-based carbon fiber epoxy composite whose parameters were experimentally determined [23]. Its dynamic characteristics were measured by boring aluminum engine blocks, and the dynamic stiffness of such a bar was increased by approximately 30%. Chatter did not occur when the ratio of length and diameter was about 30% greater than for the tungsten bar. The influence of different inner cores on the dynamic behavior and technical capabilities of non-rotating boring bars (250 mm × 25 mm) during machining of a rotating workpiece with an inner diameter of 60 mm was successfully evaluated [24]. The limit of stability depends not only on the mechanical properties of the boring bar, but also on its fixation and on the machine tool. Performance limiting machining parameters can be raised, thereby improving the productivity of machining operations. In [25] the vibration stability of the boring process is discussed, in which the workpiece rotates, a medium-size bar (0.3 m × 0.020 m) is fixed, and a passive dynamic vibration absorber is used. The boring bar is modelled as an Euler–Bernoulli cantilever beam and the absorber is considered to be attached by a spring and a damper at a certain cross-section of the beam. In order to determine the optimal values of the absorber parameters, the criterion was the maximization of the minimum values of the stability lobe diagram and the classic Nelder–Mead method was used for unconstrained optimization problems. However, no machining test was performed. The stability limit was strongly improved by increasing it by about 15% and the stability lobes were only shown analytically. Active damping of the boring bars with an in-house designed magnetic actuator offers good prospects for practical use in the case of large boring tools [26]. A boring bar with an internal friction damper has also been proposed to reduce chatter vibration of the boring bars [27]. This simple friction damper consisted of several pins axially mounted inside the bar, which caused resistance and energy dissipation during bending vibrations. Unfortunately, users of large-size boring tools have practically no possibility to modify the elastic-damping properties of these tools on their own.

A separate investigation concerns the mechanics of single-point boring operations used in the industry, due to its valuable nature in modelling the chip thickness distribution along the cutting edge [3]. Cutting forces are correlated to the chip area using mechanistic cutting force coefficients. The latter makes it possible to predict them satisfactorily in three Cartesian directions. The model is an essential foundation for examining not only chatter, but also forced vibrations in boring processes. A mathematical model of the cutting force system was developed when boring holes with large diameters and boring head with multiple inserts is spinning [28]. The model includes tool geometry, chip load, cutting edge contact length and process parameters, kinematics and mechanics of the boring process, as well as axial and radial runouts. When runout occurs in the inserts the cutting forces normal to the axis of the hole become periodic at the tooth passing frequency. The model has been experimentally verified with success, so that the surface finish and dimensional quality of the holes are maintained by avoiding excessive forced vibrations. In [29] an analytical model for the stability of turning and boring processes is proposed, which includes the dynamics of the system in a multi-dimensional form with emphasis on the true process geometry and the insert nose radius in a precise manner.

A thorough and exhaustive review of publications in the field of production engineering proves that despite many scientific reports on the dynamics of the boring process, an insufficient number is noted, especially in the case of large-size workpieces. Moreover, scientific research is far away from the requirements in this field [30]. The reason is that academia usually does not have access to the requirements of this niche industry, and the topic, as a cross-sectorial one, is not of particular interest to academia due to the limited possibilities for in-depth research. A high level of investment is also required to have large machine tools in the laboratories. Therefore, most advances in machine tools for large parts have been achieved thanks to solutions proposed by and implemented in close cooperation with the industry.

Large-scale machining is increasingly becoming the domain of small and medium-sized enterprises that do not have their own research facilities. Hence, their interests do not concern the implementation of costly integrated computerized system solutions, but the ad hoc optimization of individual technological activities at the level of process implementation. After all, the production of large-size products is usually estimated at a small number of details and due to the expected profitability—a separate approach is required for each type of product.

The above considerations indicate the only direction of effective solving of the problems of boring large-size objects, which is the performance of basic and applied research in strict cooperation with an industrial partner. The subject of the article is a method of searching for conditions for minimizing the vibration level of a rotating tool during the boring process in large-size products, based on the results of previous computer simulations of the identified boring tool model. The article in question is a creative, progressive and thorough modification of the considerations presented earlier in [31]. However, unlike the previous one, a new computational model of the boring process was proposed in the phenomenological aspect, in which:

– discrete modeling of an inherently rigid workpiece was abandoned;
– the focus was put on the discrete modeling of a boring tool with the use of a set of flexible bars, and on the assessment of model compliance with a real object;
– vibrations in the boring process were observed in all directions, and the cutting process itself shows the features of geometric nonlinearity due to the dynamic change in the thickness and width of the layer.

## 2. Simulation Model

**Figure 1:**
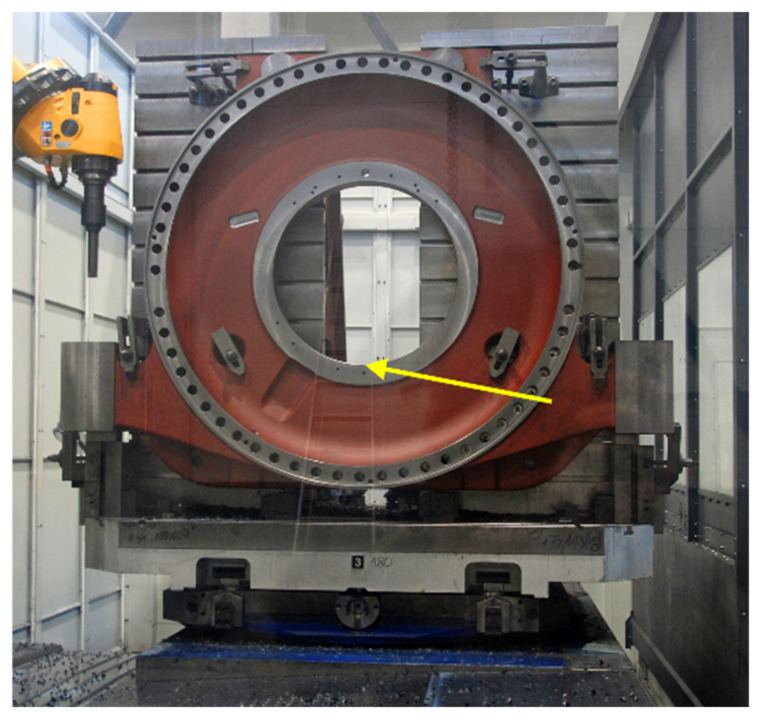
View of a large-size workpiece mounted on the table of the WHN 13-15 CNC machine. The roughness measurement point is indicated.

**Figure 2:**
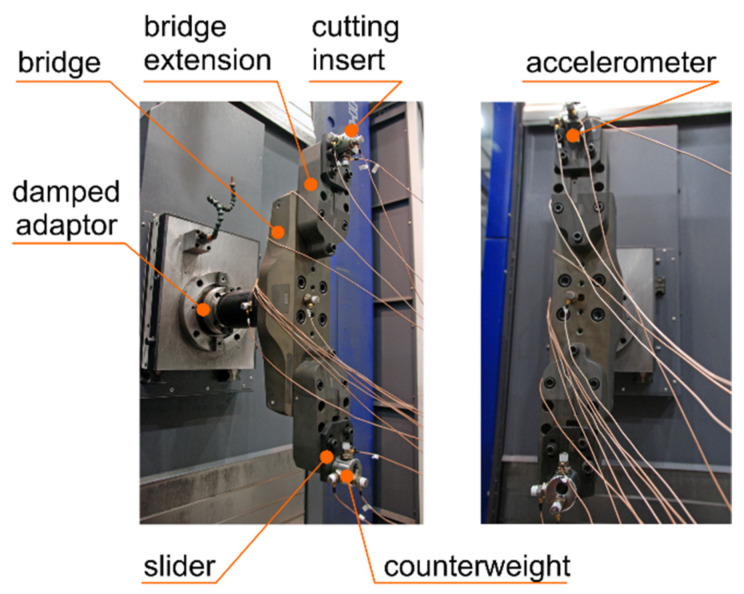
Boring tool views with attached accelerometers in the minimum extension of the damped adaptor [31].

**Figure 3:**
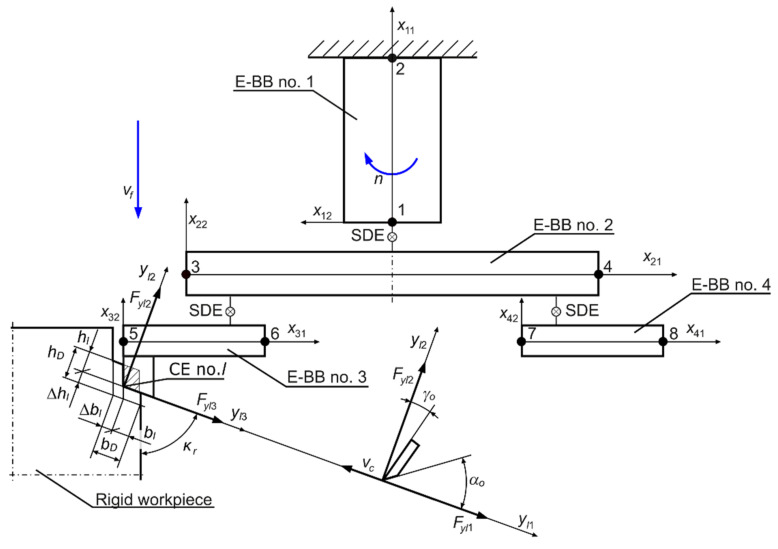
The computational model of boring process of the rigid workpiece in the FEM convention, including Euler–Bernoulli Bars (E-BB), Spring-Damping Elements (SDEs) and Coupling Element (CE) no. *l*.

The process of boring holes in a perfectly rigid large-size workpiece mounted on a machine tool table (Figure 1) using a rotating flexible boring tool (Figure 2) can be considered in the convention of a non-stationary Finite Element Model (FEM) [4,32,33]. When boring holes of large diameters, the design of the tool is more complicated and cannot be modelled with a single bar. Thus, in the appropriate machining process model (Figure 3), it is possible to distinguish structural system, i.e., non-stationary discrete model of rotating indexable tool with a desired spindle speed *n* and linearly displacing with a desired feed speed *v_f_*, as a set of 4 flexible finite elements, the Euler–Bernoulli Bars (E-BBs) [32], Spring-Damping Elements (SDEs) [34], and cutting process, i.e., Coupling Element (CE) no. *l* [32,35]. Hence, the behavior of the structural system obtained in this way, unlike the hybrid system considered earlier [31], is described by the vector of its generalized coordinates **q** in local Cartesian coordinate systems *x_e_*_1_, *x_e_*_2_, *x_e_*_3_ of every E-BB no. *e*. The relatively small (much below 1000 rpm) values of the boring tool rotational speed allow for disregarding the influence of the gyroscopic effects related to spinning on its dynamic properties [32,36].

Moreover, the scheme of the boring process shows:

– rake angle *γ*_0_ and clearance angle *α*_0_, as elements of cutting edge geometry in the orthogonal plane of the edge,
– cutting edge angle *κ_r_*,
– force *F_yl_*_1_, acting in the direction of the cutting speed *v_c_*,
– the time-varying thickness of the cutting layer *h_l_*, and the force acting in its direction—*F_yl_*_2_,
– the time-varying width of the cutting layer *b_l_*, and the force acting in its direction—*F_yl_*_3_.

Within the range of the cutting speed of 100–240 m/min and the changed depth of cutting in the range of 0.625–1.750 mm, the cutting forces in boring have a linear dependency with the chip area [3]. The latter allows for assumptions about the proportional model of the cutting process [37,38]. Moreover, the cutting forces are separated into tangential and friction directions, but the friction force is further projected into the radial and feed directions [3]. Hence, taking into account the changes in the thickness *h_l_*(*t*) and the width *b_l_*(*t*) of the cutting layer over time, the components of the cutting forces acting in the direction of the Cartesian coordinates *y_l_*_1_, *y_l_*_2_, *y_l_*_3_ of CE no. *l*, were obtained in the forms:

(1) $$F_{yl1}\left(t\right)=\left\{\begin{array}{cc} k_{dl}b_{l}\left(t\right)h_{l}\left(t\right), & h_{l}\left(t\right)>0\;˄\;b_{l}\left(t\right)>0,\\ \;\;\;\;\;\;\;\;0, & h_{l}\left(t\right)\leq 0\;˅\;b_{l}\left(t\right)\leq 0, \end{array}\right.$$

(2) $$F_{yl2}\left(t\right)=\left\{\begin{array}{cc} \mu_{l2}k_{dl}b_{l}\left(t\right)h_{l}\left(t\right), & h_{l}\left(t\right)>0\;˄\;b_{l}\left(t\right)>0,\\ \;\;\;\;\;\;\;\;0, & h_{l}\left(t\right)\leq 0\;˅\;b_{l}\left(t\right)\leq 0, \end{array}\right.$$

(3) $$F_{yl3}\left(t\right)=\left\{\begin{array}{cc} \mu_{l3}k_{dl}b_{l}\left(t\right)h_{l}\left(t\right), & h_{l}\left(t\right)>0\;˄\;b_{l}\left(t\right)>0,\\ \;\;\;\;\;\;\;\;0, & h_{l}\left(t\right)\leq 0\;˅\;b_{l}\left(t\right)\leq 0, \end{array}\right.$$

where:

$$b_{l}\left(t\right)=b_{D}-\Delta b_{l}\left(t\right)$$

$$h_{l}\left(t\right)=h_{D}-\Delta h_{l}\left(t\right)+\Delta h_{l}\left(t-\tau_{l}\right)$$

and: *b_D_*—desired cutting layer width (*b_D_* = *a_e_*/sin *κ_r_*); ∆*b_l_*(*t*)—dynamic change in cutting layer width for CE no. *l*; *h_D_*—desired cutting layer thickness (*h_D_* = *f_z_* sin *κ_r_*); ∆*h_l_*(.)—dynamic change in cutting layer thickness for CE no. *l*; *k_dl_*—average dynamic specific cutting pressure for CE no. *l*; *μ_l_*_2_, *μ_l_*_3_—cutting force ratios for CE no. *l*, respectively as the quotient of the forces *F_yl_*_2_ and *F_yl_*_1_, and the forces *F_yl_*_3_ and *F_yl_*_1_; *τ_l_*—time-delay between the same position of CE no. *l* and of CE no. *l*–1; *a_e_*—radial depth of cutting; *f_z_*—feed per tooth (*f_z_* = *v_f_*/(*nz*)); *z*—number of boring tool teeth.

Dependencies (1)–(3) show that due to the presence of nonlinear constraints in the form of inequalities, the components of cutting forces have the character of strong geometric nonlinearities.

Description of cutting forces for CE no. *l* in six-dimensional space takes the form:

(4) $$F_{l}\left(t\right)=F_{l}^{0}-D_{Pl}\left(\Delta b_{l}\left(t\right)\right)\Delta w_{l}\left(t\right)+D_{Ol}\left(\Delta b_{l}\left(t\right)\right)\Delta w_{l}\left(t-\tau_{l}\right),$$

where:

(5) $$F_{l}\left(t\right)=col\left(F_{yl1}\left(t\right),F_{yl2}\left(t\right),F_{yl3}\left(t\right),0_{3\times 1}\right),$$

(6) $$F_{l}^{0}=col\left(k_{dl}b_{D}h_{D},\mu_{l2}k_{dl}b_{D}h_{D},\mu_{l3}k_{dl}b_{D}h_{D},\;0_{3\times 1}\right),$$

(7) $$D_{Pl}\left(\Delta b_{l}\left(t\right)\right)=\left[\begin{array}{cccc} 0 & k_{dl}\left(b_{D}-\Delta b_{l}\left(t\right)\right) & k_{dl}h_{D} & \\ 0 & \mu_{l2}k_{dl}\left(b_{D}-\Delta b_{l}\left(t\right)\right) & \mu_{l2}k_{dl}h_{D} & 0_{3\times 3}\\ 0 & \mu_{l3}k_{dl}\left(b_{D}-\Delta b_{l}\left(t\right)\right) & \mu_{l3}k_{dl}h_{D} & \\ & 0_{3\times 3} & & 0_{3\times 3} \end{array}\right],$$

(8) $$D_{Ol}\left(\Delta b_{l}\left(t\right)\right)=\left[\begin{array}{cccc} 0 & k_{dl}\left(b_{D}-\Delta b_{l}\left(t\right)\right) & 0 & \\ 0 & \mu_{l2}k_{dl}\left(b_{D}-\Delta b_{l}\left(t\right)\right) & 0 & 0_{3\times 3}\\ 0 & \mu_{l3}k_{dl}\left(b_{D}-\Delta b_{l}\left(t\right)\right) & 0 & \\ & 0_{3\times 3} & & 0_{3\times 3} \end{array}\right],$$

(9) $$\Delta w_{l}\left(t\right)=col\left(q_{zl}\left(t\right),\Delta h_{l}\left(t\right),\Delta b_{l}\left(t\right),0_{3\times 1}\right),$$

(10) $$\Delta w_{l}\left(t-\tau_{l}\right)=col\left(q_{zl}\left(t-\tau_{l}\right),\Delta h_{l}\left(t-\tau_{l}\right),\Delta b_{l}\left(t-\tau_{l}\right),0_{3\times 1}\right),$$

and: $q_{zl}\left(t\right)$—relative displacement of edge tip and workpiece along direction *y_l_*_1_ at instant of time *t*; $q_{zl}\left(t-\tau_{l}\right)$—relative displacement of edge tip and workpiece along direction *y_l_*_1_ at instant of time *t—τ_l_*.

Components of generalized displacements $\Delta w_{l}\left(t\right)$ in the Cartesian coordinate system *y_l_*_1_, *y_l_*_2_, *y_l_*_3_ of CE no. *l* are related to the vector of generalized displacements **q** of the structural system using the time-dependent constraints equation [32]:

(11) $$\Delta w_{l}\left(t\right)=T_{l}\left(t\right)q\left(t\right),$$

where: **T***_l_*(*t*)—transformation matrix of displacements vector **q** from the *x_e_*_1_, *x_e_*_2_, *x_e_*_3_ coordinates of E-BBs, *e* = 1, …, 4, to the coordinate system *y_l_*_1_, *y_l_*_2_, *y_l_*_3_ of CE no. *l* [32,35,39].

The equation of the dynamics of the non-stationary model of the cutting process, after taking into account the expression (11), has the final form:

(12) $$M\ddot{q}+L\dot{q}+\left(K+\sum_{l=1}^{i_{l}}T_{l}^{T}\left(t\right)D_{Pl}\left(q\right)T_{l}\left(t\right)\right)q=\sum_{l=1}^{i_{l}}T_{l}^{T}\left(t\right)F_{l}^{0}+\sum_{l=1}^{i_{l}}T_{l}^{T}\left(t\right)D_{Ol}\left(q\right)\Delta w_{l}\left(t-\tau_{l}\right),$$

where *:*M, L, K—matrices of inertia, damping and stiffness of the set of E-BBs and accompanying SDEs, *i_l_*—number of “active” coupling elements, i.e., cutting edges currently being in contact with the workpiece. The way of determining these matrices is shown in the Appendix A. The dependence of the matrices $D_{Pl}$ and $D_{Ol}$ on the vector of generalized displacements q results in another argument proving the geometric nonlinearity of the model of dynamics of the boring process, described by Equation (12). It should be noted that the latter equation describes the behavior of the structural system in generalized coordinates and shows significant advantages over the description of the behavior of the system considered in hybrid coordinates [31].

## 3. Selecting the Best Spindle Speed

**Figure 4:**
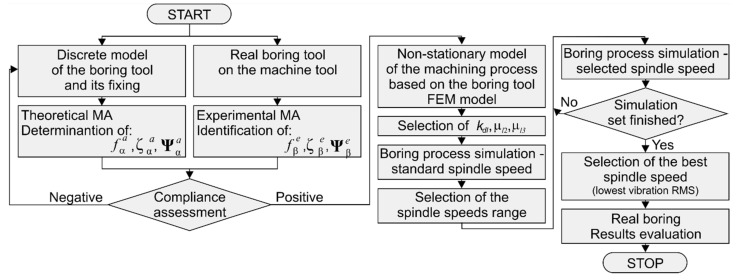
Scheme of choosing the best spindle speed in the process of boring large-sized workpieces using E-AVP. The scheme is based on [31].

The proposed method of supervising the boring process by Experiment-Aided Virtual Prototyping (E-AVP) determines the best rotational speed of the tool based on the simulation of the boring process, carried out for a computational model of the tool tuned to the results of modal tests, along with the adopted model of the cutting process (Figure 4).

The FEM parameters of the boring tool obtained by the Theoretical Modal Analysis (TMA) were validated with the results of the Experimental Modal Analysis (EMA). The validation concerned the natural frequencies of the dominant vibration modes *f_α_*, dimensionless damping coefficients $\zeta_{\alpha }$ and vectors of normal modes $\Psi_{\alpha }$. It should be noted that the frequently used Modal Assurance Criterion (MAC) [4,32] was not applied here. The reason is that the positions of the E-BBs nodes are, in general, different from the locations of the accelerometers used in the experiment. After taking into account the time-varying positions of the edges (CE no. *l*) of the boring tool, a non-stationary computational model of the machining process is created. It is impossible to estimate the parameters of the computational model of the boring process based on the assessment of compliance of the Root Mean Square (RMS) of the simulated plots with the values obtained during the measurements in the real process. However, due to the simplicity of the mathematical description of the adopted model of proportional cutting dynamics, which is its advantage, for the correct estimation of the parameters of the cutting process, it is enough to actually choose only 3 values of abstract meaning, i.e., average dynamic specific cutting pressure *k_dl_* and the cutting force coefficients *μ*_2_ and *μ*_3_. Determining the values of these parameters is only a means to an end. The exact determination of the values sought is time-consuming, and at the same time does not significantly translate into improved results. Hence, these cutting process parameters are estimated using the so-called “mechatronic” procedure [40]. It allows, in contrast to the method presented in [4], to resign from the simulation cycle for the case of cutting with the standard spindle speed in order to adjust the values of these parameters. It is sufficient to perform a single simulation, the results of which form the basis for the selection of the best speed later. Subsequently, the permissible range of tested spindle speeds is selected and for chosen speeds from this range, the cutting process simulations are performed. It should be noted that instead of the previously considered abstract hybrid model [31], this time a non-stationary structural model of the machining process with deep physical meaning was used for the simulation. The spindle speed is selected as the best if it assures the lowest vibration level or the lowest dominant amplitudes in the spectra.

The key elements of the proposed method are the identification of model parameters of the boring tool itself (before machining), estimation of the necessary parameters of the cutting process, computer simulations made for a finite set of spindle speeds, and finally the machining process with parameters derived from the simulation results. It is an innovative model of decision support in the design of boring processes in large-size objects and is addressed to a wide group of recipients interested in the results of research on the quality and efficiency of production.

## 4. Modal Identification of Boring Tool

**Figure 5:**
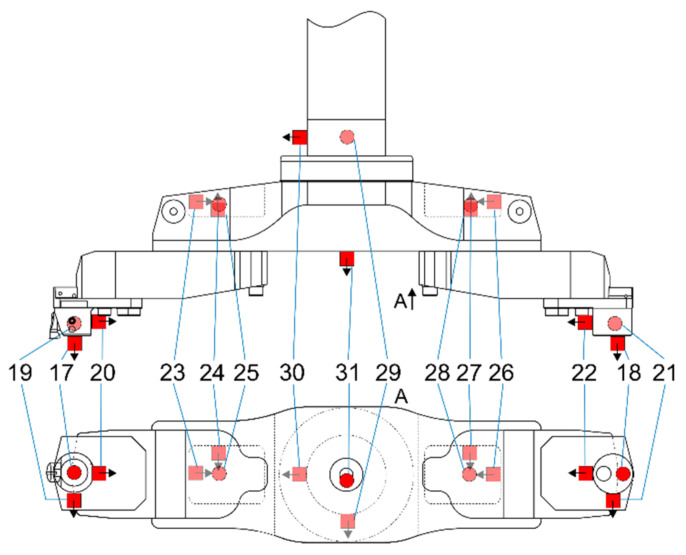
Scheme of the Sandvik CoroBore 825 XL boring tool with marked 1-axis accelerometers positions. Black arrows indicate the positive direction of measured accelerations. Accelerometers marked with a lighter color are covered by the object [31].

During the experimental investigation, the Sandvik CoroBore^®^ 825 XL boring tool, manufactured by Sandvik Coromant, Sandviken, Sweden, an international supplier of the cutting tools was used [41]. Modal identification of the boring tool mounted in a damped adaptor at its minimal extension (see, Figure 2) was performed in order to validate the parameters of the tool FEM, in accordance with the procedure presented in the previous section. Positions and directions of the accelerometers used during modal tests are shown in Figure 5. Impact modal tests were performed with a modal hammer and the object responses were measured with 15 accelerometers (marked as A17–A31 in Figure 5). There were 4 sets of 40 impacts made in the direction of accelerometers A18, A21, A22 and A29. For each set, the Frequency Response Function (FRF) was calculated using the H3 estimator.

**Figure 6:**
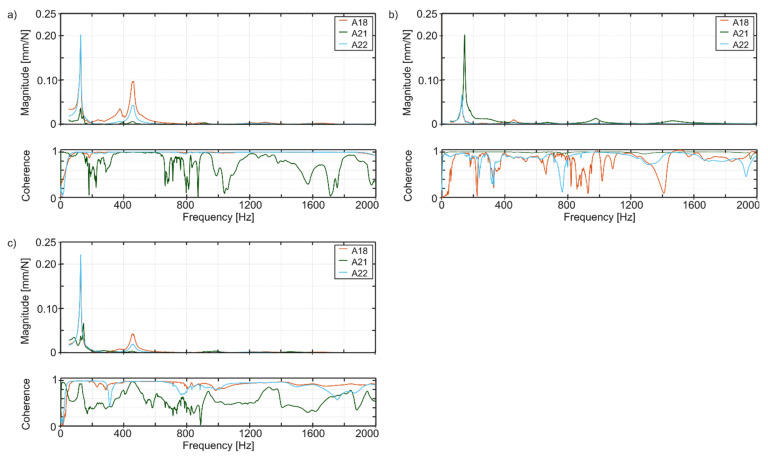
Exemplary force-displacement FRFs of the boring tool for excitation in the direction of sensor no. (**a**) A18, (**b**) A21, (**c**) A22.

Examples of the FRF are illustrated in Figure 6. From the point of view of the analysis of vibrations occurring during machining, the most important are the modes of vibration in the low-frequency range, especially those characterized by significant values of displacement. For the tested boring tool, the frequencies up to 2000 Hz were analyzed. One should also pay attention to the coherence function. Seemingly, it looks low on the collective chart. However, if you look at individual channels, it turns out that the low values of coherence relate primarily to those channels that were weakly excited at a given point. For example, when excitation was acting in the direction of A18, low coherence concerns accelerometers A22 and, especially, A21, which measure accelerations in planes perpendicular to excitation direction. Figure 6 shows FRFs measured only for A18, 21 and 22 to make plots easier to analyze. During tests, FRFs for all of the 15 accelerometers were calculated and later used for modal identification.

Identification of the parameters of the modal model of the boring tool was performed using the ERA (*Eigenvalue Realisation Algorithm*) [4,32,42] and the p-LSCFD (*polyreference—Least Squares Complex Frequency Domain*) [32,43,44] methods. Measurements were made by uniaxial accelerometers that were mounted to measure vibration for three orthogonal directions at 5 different points (Figure 5). An important element facilitating the assessment of the correctness of the estimated figures is the symmetrical configuration of the structure. The displacements on one side of the boring tool should correspond to the displacements on the other side.

The values of the natural frequencies and dimensionless damping coefficients (the so-called modal damping), determined using both methods in the frequency range from 0 to 2000 Hz, are summarized in Table 1. The convergence of their values proves the correctness of the identified modal parameters.

**Figure 7:**
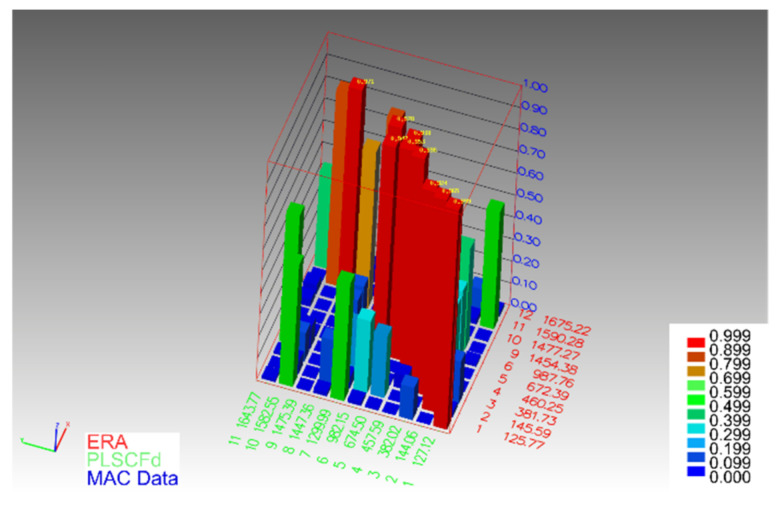
MAC values for normal modes obtained by ERA and p-LSCFD methods.

The determined normal modes of natural vibrations in the frequency range up to 1000 Hz, using both identification methods, are of very good quality. This is evidenced by the comparison of the results of both methods with the use of the MAC criterion (Figure 7).

**Figure 8:**
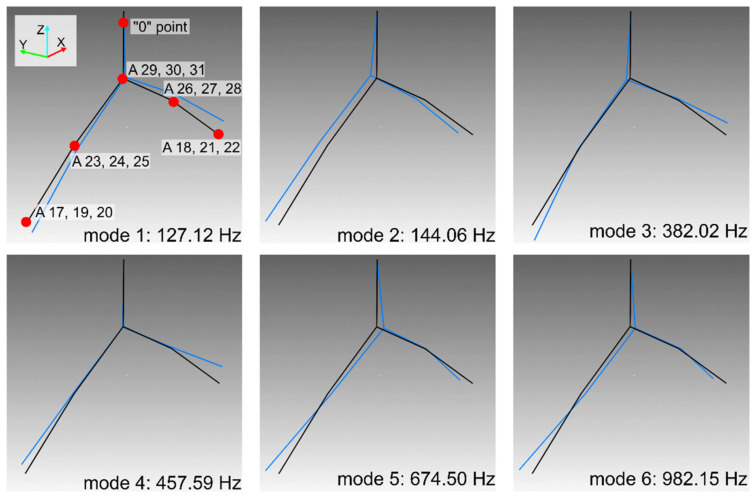
Normal modes of vibration of a boring tool determined by the p-LSCFD method. For the mode 1, sensor locations are marked.

Graphs of the normal modes of the boring tool in the frequency range up to 1000 Hz are presented using the wireframe model in Figure 8. The presented modes are normalized in such a way that for each mode the maximum deflection from the equilibrium position is equal to one. The tool holder is located in the upper part of the drawing. The first two figures illustrate bending modes with respect to clamping the boring tool in the *X*-*Z* and *Y*-*Z* planes. The third form is the bending of the boring tool itself in the *X*-*Z* plane. The fourth figure is a “winglet” mode. The fifth and sixth forms are very similar and are bending ones in the *Y*-*Z* plane. The first torsional mode has not been identified, which may be due to its effective damping by the propulsion system.

## 5. The Calculation Model of a Boring Tool

Based on the characteristic geometrical dimensions of the CoroBore^®^ 825 XL boring tool, a FEM was created containing four 2-node finite elements of the Euler–Bernoulli Bar (E-BB) type (6 degrees of freedom in each node), connected by Spring-Damping Elements (SDE) [31,32] (see, Figure 3).

**Figure 9:**
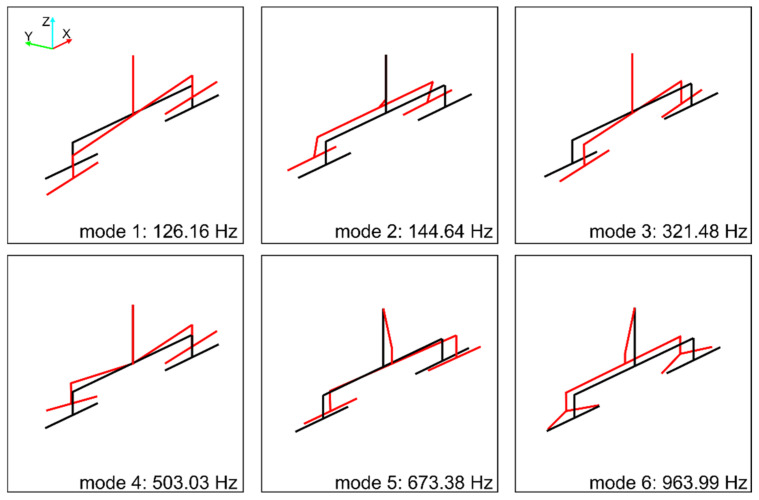
Normal modes of vibration of a boring tool determined by the calculation model.

Using the proprietary programs developed in Fortran, the frequencies and modes of natural vibrations of the created computational model were calculated. The calculation results for the first 6 normal modes are graphically illustrated in Figure 9. These results were compared with the boring tool modal parameters obtained from the identification (Table 1, Figure 8). Despite slight differences in the values of frequencies 3 and 4, the obtained results were found to be consistent. This also applies to the calculated modes of natural vibrations. With the exception of some differences, mainly noticeable in forms 3rd and 5th, in other cases, the obtained figures generally reflect the image consistent with the experimentally determined. The above allows us to take into account the obtained computational model of the boring tool in computer simulations of the boring process.

## 6. Computer Simulations

The process of boring a hole (∅ 733.44 mm) in rigid large-size cast iron (EN-GJS-400-18-LT) workpiece, from the standard production program of the industrial partner PHS HYDROTOR Inc., Tuchola, Poland, was simulated using proprietary software developed in Fortran. Taking into account the previously postulated “mechatronic” procedure [40], on the basis of the considerations presented in the literature [3], the values of the parameters of the calculation model of the boring process were estimated as: *k_dl_* = 20 daN/mm^2^, *µ_l_*_2_ = 0.4, *µ_l_*_3_ = 0.2. Selected simulation results for 5 different pairs of technological parameter values are summarized in Table 2. Contrary to the considerations presented in [31], this time the vibrations were observed in three characteristic directions of the components of the cutting force [3], i.e., radial, feed speed and cutting speed. Thanks to creative modifications of the simulation software, the average simulation time was reduced to about 20% of the main machining time in the case of the simulation run on the Intel Core i7 6700 processor, compared to the calculations in [31].

The machining was first considered according to the standard technology of the cooperating company (i.e., *n* = 105 rpm, *v_f_* = 9.6 mm/min), and then the obtained results were compared with the proposed method. Then, the hole was machined for standard parameters, and subsequently—in a series of repetitive simulations—the predicted values of the best spindle speeds were determined, for which the boring process was planned to be carried out.

**Figure 10:**
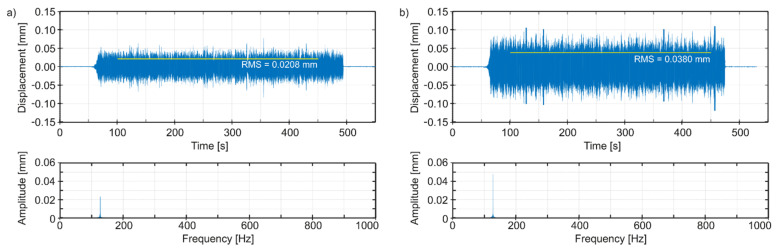
Simulated vibrations of the edge tip in the radial direction: (**a**) the best spindle speed *n* = 120 rpm, (**b**) adverse spindle speed *n* = 125 rpm.

**Figure 11:**
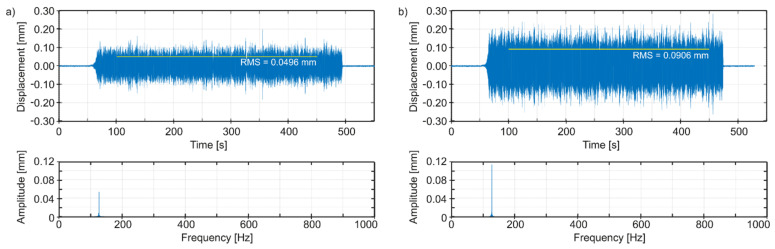
Simulated vibrations of the edge tip in the feed speed direction: (**a**) the best spindle speed *n* = 120 rpm, (**b**) adverse spindle speed *n* = 125 rpm.

**Figure 12:**
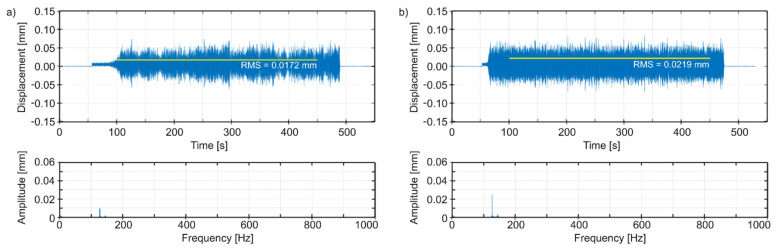
Simulated vibrations of the edge tip in the cutting speed direction: (**a**) the best spindle speed *n* = 115 rpm, (**b**) adverse spindle speed *n* = 125 rpm.

Thus, in Figure 10a, we can observe time plots and spectra of vibrations of the tool edge tip in the radial direction (i.e., normal to the bored hole surface), for the best spindle speed, and in Figure 10b for an adverse spindle speed. In Figure 11a, we can see time plots and spectra of vibrations of the tool edge tip in the feed speed direction, for the best spindle speed, and in Figure 11b for an adverse spindle speed. In Figure 12a, we can observe time plots and spectra of vibrations of the tool edge tip in the cutting speed direction, for the best spindle speed, and in Figure 12b for an adverse spindle speed. The starting times of the observation of the spectra were 300 s, with 2^17^ samples for each plot. It is easy to see that at an adverse spindle speed, the vibrations that occur are much greater. In addition, relatively low excitation frequencies from 1.75 to 2.08 Hz following the entry of the cutting edge into the material and resulting from the adopted range of rotational speeds of the boring tool, cause—in all cases—vibrations to be stimulated with a frequency close to the first mode of natural vibrations, with the predominant vibration amplitudes in the feed speed direction.

The Root Mean Square (RMS) values and amplitudes of dominant peaks (*q*_0_) in spectra are evaluated for various values of technological parameters. Comparing the simulation results (Table 2) in the radial direction and in the feed speed direction, we obtain the best configuration of parameters for *n* = 120 rpm, but the adverse configuration of parameters for *n* = 125 rpm. Although in the cutting speed direction, the best configuration of parameters is obtained for *n* = 115 rpm, the direction of these vibrations, unlike the first two, is not decisive from the point of view of machining quality (geometric accuracy, surface roughness). Hence, it can be assumed that the best-simulated spindle speed is *n* = 120 rpm.

## 7. Experimental Research of the Boring Process

Experimental research to evidence the accuracy of computer predictions, concerned boring a hole in a rigid large-size cast iron (EN-GJS-400-18-LT) workpiece, which was mounted on a milling and boring WHN 13-15 CNC machine, produced by TOS VARNSDORF A.S. in Varnsdorf, Czech Republic (see, Figure 1). Boring was performed using Sandvik CoroBore^®^ 825 XL boring tool (see, Figure 2 and Figure 5). In Table 3, the roughness parameters R_a_ and R_z_ of the hole surface at the lowest place (Figure 1) are compared, obtained in accordance with the standard technology of the cooperating industry and on the basis of 3 modifications of technological parameters, as a consequence of previously conducted simulations (Table 2).

Compared to the standard technology (i.e., *n* = 105 rpm, *v_f_* = 9.6 mm/min), the vibration level decreased noticeably in the case of the best simulation (Table 2). The RMS values were reduced accordingly: in the radial direction—by 9%, in the feed speed direction—by 9%, and in the cutting speed direction—by 4%. The dominant amplitudes in the spectra remained almost the same comparing to standard machining technology. In addition, the evident reduction in vibration of the boring tool is accompanied by an increase in the spindle speed from 105 to 120 rpm (i.e., 14%), which results in a reduction of the main machining time by 0.89 min (11%) and a simultaneous increase in machining efficiency. At the same time, the measured values of the *R_a_* and *R_z_* parameters did not deteriorate (Table 3), which confirms that the requirements for maintaining the required surface quality were assured.

Although a further increase in the spindle speed to 125 rpm results in a reduction of the roughness parameters, doing so is in fact not recommended. This is because it adversely affects the durability of the cutting inserts, significantly shortening their service life. Thereby, it unreasonably increases processing costs.

## 8. Implementation Profitability Assessment

The purpose of these considerations, which make a significant contribution compared to [31], is to develop the basis for managerial insights that facilitate the accuracy of decision-maker actions in the industrial practice of small and medium-sized enterprises that are not interested in implementing advanced system solutions, but in meeting the requirements of the profitability criterion at the technological process level of the product.

In order to obtain information on the profitability resulting from the application of the presented method, the standards of technological times were compared [45]. Let us reconsider the process of boring a hole in a rigid large-size cast iron EN-GJS-400-18-LT workpiece on the WHN 13-15 CNC table milling and boring machine in one pass. According to the standard technology, such machining has so far been performed in a unit time (approximately equal to the execution time), which is:

*t_j_*_0_ = *l_p_*_0_ × *t_pk_*_0_ + *t_g_*_0_ (13)

where:

– *l_p_*_0_—number of inspection cuts for pass,
– *t_pk_*_0_—time of inspection cut of pass,
– *t_g_*_0_—main time of pass.

For the sake of simplicity, in Formula (13), other components of the auxiliary time *t_p_* were omitted. Assuming *l_pk_*_0_ = 1, *t_pk_*_0_ = *t_g_*_0_ = 8.02 min., we get *t_j_*_0_ = 16.04 min.

In the solution for the best combination of technological parameters, the expected main time of pass is *t_g_* = 7.13 min., and there are no inspection passes (*l_p_* = 0). This gives the total unit time *t_j_* = 7.13 min, which is 56% shorter. The total time per unit for boring a hole is therefore:

– for standard technology—16.04 min,
– in the case of technology based on the best combination of parameters—7.13 min.

In the case of the proposed technology, there is no need to increase the machining time by the theoretical and experimental modal analysis times, and by the boring simulation series time, because the above concern only the boring tool, regardless of the number of rigid workpieces mounted on the machine.

The profitability of applying the obtained results is therefore seen in the category of:

– minimizing the vibration level of the boring tool, thereby maintaining the required product quality, and
– shortening the total standard of time by 8.91 min, i.e., by 56%.

## 9. Conclusions

The results obtained during the research confirmed, in the production conditions of the industrial partner, the effectiveness of the proposed method of suppressing the vibrations of a rotating boring tool. Because of the use of the Experiment-Aided Virtual Prototyping (E-AVP), the best spindle speed was selected when boring holes in large-size structures. Due to technical impossibility, the vibrations of the workpiece were not measured on-line during machining. Nevertheless, the results of roughness measurements (i.e., *R_a_*, *R_z_*) of the bored surface made with a profilometer after boring confirmed the accuracy of the prediction for the best selection of technological parameters *n*, *v_f_* for the machining process.

The present study makes it possible to predict the best conditions of the process of boring large workpieces only on the basis of a simulation of the computational model of the process in which the parameters of the boring tool were validated with a real object. The approach presented in the article, due to its uncomplicated nature, can be easily applied in the economic practice of many companies dealing with the machining of large-size objects—even those who do not have their own research facilities and their own hardware and software infrastructure.

The applied method does not require interference in the structure of the machine tool and, apart from identifying the computational model of the boring tool, no previous experimental tests are needed to simulate the boring process. Computational models of many boring tools can be prepared off-line for the selected configurations (e.g., overhang, boring diameter), and then the appropriate model can be selected to perform the simulation.

The assessment of the profitability of implementing the proposed method should be seen in the category of minimizing the vibration level of the boring tool, resulting in an improvement in the quality of workmanship, as well as a significant reduction in the production standard of the unit execution time. In addition, the cost reduction of the material to be removed cannot be overestimated due to the lack of inspection cuts that actually exist in standard technology.

Although the increased efficiency of boring holes in large-size workpieces was accompanied by the maintenance of roughness indicators appropriate for finishing, research perspectives should be directed at the successive improvement of the quality of the machined surface, obtaining *R_a_* below 1 µm. This challenge is to be met by the method of suppressing the vibration level of a rotating tool proposed in this paper, effectively implemented at the level of the boring process.

## Figures and Tables

**Table 1:** Natural frequencies and modal damping coefficients of the identified normal modes of CoroBore 825 XL boring tool.

Mode Number	ERA Natural Frequency (Hz)	ERA Modal Damping (%)	Mode Number	p-LSCFD Natural Frequency (Hz)	p-LSCFD Modal Damping (%)
1	125.77	3.3	1	127.12	2.7
2	145.59	4.0	2	144.06	2.8
3	381.73	2.7	3	382.02	2.8
4	460.25	2.6	4	457.59	2.6
5	672.39	3.6	5	674.50	3.6
6	987.76	2.1	6	982.15	1.9
7	1128.6	1.5	–	1130.7	2.1
8	1203.8	3.5	–	1222.8	2.6
–	–	–	7	1299.99	3.1
9	1454.38	2.0	8	1447.36	1.7
10	1477.27	2.3	9	1475.39	2.5
11	1580.28	2.1	10	1582.56	1.9
12	1675.22	1.9	11	1643.77	2.5

**Table 2:** Simulated vibrations displacements of the edge tip. The RMS values and dominant peaks of amplitudes in spectra. Underlined value—the best configuration, bold value—adverse configuration.

Spindle Speed	Feed Speed	1st Natural Frequency	Radial Direction		Feed Speed Direction		Cutting Speed Direction	
*n* (rpm)	*v_f_* (mm/min)	*f* (Hz)	RMS (mm)	*q*_0_ (mm)	RMS (mm)	*q*_0_ (mm)	RMS (mm)	*q*_0_ (mm)
105	9.6	127.21	0.0229	0.0234	0.0546	0.0535	0.0196	0.0180
110	10.1	126.26	0.0360	0.0390	0.0858	0.0930	0.0187	0.0162
115	10.5	126.26	0.0323	0.0250	0.0770	0.0597	0.0172	0.0104
120	11.0	127.35	0.0208	0.0233	0.0496	0.0535	0.0189	0.0197
125	11.4	126.65	**0.0380**	**0.0475**	**0.0906**	**0.1133**	**0.0219**	**0.0248**

**Table 3:** Parameters and results of boring the large-size workpiece [31].

Procedure	*a_e_* (mm)	*n* (rpm)	*v_f_* (mm/min)	*D* (mm)	*R_a_* (µm)	*R_z_* (µm)	Technology
W1	1	105	9.6	727.4632	5.080	24.73	Standard
W2	1	110	10.1	729.4516	4.063	19.93	Modification 1
W3	1	120	10.8	731.4521	5.852	26.53	Modification 2
W4	1	125	11.9	733.4432	3.935	20.09	Modification 3

## Data Availability

The data presented in this study are openly available at MostWiedzy as: Galewski M.A., Mazur M.R., Kaliński K.J., Frequency Response Functions for Sandvik CoroBore 825 XL boring tool, 733 mm [Data set]. Gdańsk University of Technology, 2021, https://doi.org/10.34808/nhsy-hy21.

## Appendix A

### Appendix A.1. Matrix of Shape Functions of E-BB No. e

(A1) $$N_{e}\left(\xi \right)={\left[\begin{array}{cc} \begin{array}{cc} N_{e11} & N_{e12}\\ N_{e21} & N_{e22} \end{array} & \begin{array}{cc} N_{e13} & N_{e14}\\ {-N}_{e21} & N_{e24} \end{array} \end{array}\right]}_{6\times 12}$$

where:

(A2) $$N_{e11}=\left[\begin{array}{ccc} 1-\xi & -x_{e2}\cdot \frac{6}{l_{e}}\xi \left(\xi -1\right) & x_{e3}\cdot \frac{6}{l_{e}}\xi \left(1-\xi \right)\\ 0 & 2\xi^{3}-3\xi^{2}+1 & 0\\ 0 & 0 & 2\xi^{3}-3\xi^{2}+1 \end{array}\right],$$

(A3) $$N_{e12}=\left[\begin{array}{ccc} 0 & x_{e3}\cdot \left(3\xi^{2}-4\xi +1\right) & -x_{e2}\cdot \left(3\xi^{2}-4\xi +1\right)\\ -x_{e3}\cdot \left(1-\xi \right) & 0 & \xi l_{e}\left(\xi^{2}-2\xi +1\right)\\ x_{e2}\cdot \left(1-\xi \right) & \xi l_{e}\left(-\xi^{2}+2\xi -1\right) & 0 \end{array}\right],$$

(A4) $$N_{e13}=\left[\begin{array}{ccc} \xi & -x_{e2}\cdot \frac{6}{l_{e}}\xi \left(1-\xi \right) & x_{e3}\cdot \frac{6}{l_{e}}\xi \left(\xi -1\right)\\ 0 & \xi^{2}\left(-2\xi +3\right) & 0\\ 0 & 0 & \xi^{2}\left(-2\xi +3\right) \end{array}\right],$$

(A5) $$N_{e14}=\left[\begin{array}{ccc} 0 & x_{e3}\cdot \left(3\xi^{2}-2\xi \right) & -x_{e2}\cdot \left(3\xi^{2}-2\xi \right)\\ -x_{e3}\cdot \xi & 0 & \xi^{2}l_{e}\left(\xi -1\right)\\ x_{e2}\cdot \xi & \xi^{2}l_{e}\left(-\xi +1\right) & 0 \end{array}\right],$$

(A6) $$N_{e21}=\left[\begin{array}{ccc} 0 & 0 & 0\\ 0 & 0 & -\frac{6}{l_{e}}\xi \left(\xi -1\right)\\ 0 & \frac{6}{l_{e}}\xi \left(\xi -1\right) & 0 \end{array}\right],$$

(A7) $$N_{e22}=\left[\begin{array}{ccc} 1-\xi & 0 & 0\\ 0 & 3\xi^{2}-4\xi +1 & 0\\ 0 & 0 & 3\xi^{2}-4\xi +1 \end{array}\right],$$

(A8) $$N_{e24}=\left[\begin{array}{ccc} \xi & 0 & 0\\ 0 & \xi \left(3\xi -2\right) & 0\\ 0 & 0 & \xi \left(3\xi -2\right) \end{array}\right],$$

$\xi =\frac{x_{e1}}{l_{e}}$, $l_{e}$—length of E-BB no. *e*.

### Appendix A.2. Inertia Matrix of E-BB No. e

(A9) $$M_{e}=\rho_{e}l_{e}{\left[\begin{array}{cc} \begin{array}{cc} M_{e11} & M_{e12}\\ M_{e12}^{T} & M_{e22} \end{array} & \begin{array}{cc} M_{e13} & M_{e14}\\ M_{e23} & M_{e24} \end{array}\\ \begin{array}{cc} M_{e13}^{T} & M_{e23}^{T}\\ M_{e14}^{T} & M_{e24}^{T} \end{array} & \begin{array}{cc} M_{e11} & -M_{e12}\\ -M_{e12}^{T} & M_{e22} \end{array} \end{array}\right]}_{12\times 12}$$

where:

(A10) $$M_{e11}=\left[\begin{array}{ccc} \frac{A_{e}}{3} & 0 & 0\\ 0 & \frac{13A_{e}}{35}+\frac{6I_{xe3}}{5l_{e}^{2}} & 0\\ 0 & 0 & \frac{13A_{e}}{35}+\frac{6I_{xe2}}{5l_{e}^{2}} \end{array}\right]$$

(A11) $$M_{e12}=\left[\begin{array}{ccc} 0 & 0 & 0\\ 0 & 0 & \frac{11A_{e}l_{e}}{210}+\frac{I_{xe3}}{10l_{e}}\\ 0 & -\frac{11A_{e}l_{e}}{210}-\frac{I_{xe2}}{10l_{e}} & 0 \end{array}\right],$$

(A12) $$M_{e13}=\left[\begin{array}{ccc} \frac{A_{e}}{6} & 0 & 0\\ 0 & \frac{9A_{e}}{70}-\frac{6I_{xe3}}{5l_{e}^{2}} & 0\\ 0 & 0 & \frac{9A_{e}}{70}-\frac{6I_{xe2}}{5l_{e}^{2}} \end{array}\right],$$

(A13) $$M_{e14}=\left[\begin{array}{ccc} 0 & 0 & 0\\ 0 & 0 & -\frac{13A_{e}l_{e}}{420}+\frac{I_{xe3}}{10l_{e}}\\ 0 & \frac{13A_{e}l_{e}}{420}-\frac{I_{xe2}}{10l_{e}} & 0 \end{array}\right],$$

(A14) $$M_{e22}=\left[\begin{array}{ccc} \frac{I_{xe1}}{3} & 0 & 0\\ 0 & \frac{A_{e}l_{e}^{2}}{105}+\frac{2I_{xe2}}{15} & 0\\ 0 & 0 & \frac{A_{e}l_{e}^{2}}{105}+\frac{2I_{xe3}}{15} \end{array}\right],$$

(A15) $$M_{e23}=\left[\begin{array}{ccc} 0 & 0 & 0\\ 0 & 0 & -\frac{13A_{e}l_{e}}{420}+\frac{I_{xe2}}{10l_{e}}\\ 0 & \frac{13A_{e}l_{e}}{420}-\frac{I_{xe3}}{10l_{e}} & 0 \end{array}\right],$$

(A16) $$M_{e24}=\left[\begin{array}{ccc} \frac{I_{xe1}}{6} & 0 & 0\\ 0 & -\frac{A_{e}l_{e}^{2}}{140}-\frac{I_{xe2}}{30} & 0\\ 0 & 0 & -\frac{A_{e}l_{e}^{2}}{140}-\frac{I_{xe3}}{30} \end{array}\right]\;,$$

$\rho_{e}$—mass density of the E-BB no. *e* material; $A_{e}$—cross section area of E-BB no. *e*; $I_{xe1}$—second moment of area with respect to the $x_{e1}$ axis of E-BB no. *e*; $I_{xe2}$—second moment of area with respect to the $x_{e2}$ axis of E-BB no. *e*; $I_{xe3}$—second moment of area with respect to the $x_{e3}$ axis of E-BB no. *e*.

### Appendix A.3. Stiffness Matrix of E-BB No. e

(A17) $$K_{e}=\frac{E_{e}}{l_{e}}{\left[\begin{array}{cc} \begin{array}{cc} K_{e11} & K_{e12}\\ K_{e12}^{T} & K_{e22} \end{array} & \begin{array}{cc} -K_{e11} & K_{e12}\\ K_{e23} & K_{e24} \end{array}\\ \begin{array}{cc} -K_{e11}^{T} & K_{e23}^{T}\\ K_{e12}^{T} & K_{e24}^{T} \end{array} & \begin{array}{cc} K_{e11} & -K_{e12}\\ -K_{e12}^{T} & K_{e22} \end{array} \end{array}\right]}_{12\times 12}\;,$$

where:

(A18) $$K_{e11}=\left[\begin{array}{ccc} A_{e} & 0 & 0\\ 0 & \frac{12I_{xe3}}{l_{e}^{2}} & 0\\ 0 & 0 & \frac{12I_{xe2}}{l_{e}^{2}} \end{array}\right]\;,$$

(A19) $$K_{e12}=\left[\begin{array}{ccc} 0 & 0 & 0\\ 0 & 0 & \frac{6I_{xe3}}{l_{e}}\\ 0 & -\frac{6I_{xe2}}{l_{e}} & 0 \end{array}\right]$$

(A20) $$K_{e22}=\left[\begin{array}{ccc} \frac{I_{xe1}}{2\left(1+\nu_{e}\right)} & 0 & 0\\ 0 & 4I_{xe2} & 0\\ 0 & 0 & 4I_{xe3} \end{array}\right]$$

(A21) $$K_{e23}=\left[\begin{array}{ccc} 0 & 0 & 0\\ 0 & 0 & \frac{6I_{xe2}}{l_{e}}\\ 0 & -\frac{6I_{xe3}}{l_{e}} & 0 \end{array}\right],$$

(A22) $$K_{e24}=\left[\begin{array}{ccc} -\frac{I_{xe1}}{2\left(1+\nu_{e}\right)} & 0 & 0\\ 0 & 2I_{xe2} & 0\\ 0 & 0 & 2I_{xe3} \end{array}\right],$$

*E_e_*—Young modulus of the E-BB no. *e* material, $\nu_{e}$—Poisson’s ratio of the E-BB no. *e* material.

### Appendix A.4. Damping Matrix of E-BB No. e

(A23) $$L_{e}=\frac{\eta_{e}}{E_{e}}K_{e}$$

where: $\eta_{e}$—damping constant of the E-BB no. *e* material.

### Appendix A.5. Inertia Matrix of the Whole M-Degree-of-Freedom Discrete System

(A24) $$M=\overset{i_{e}}{\underset{e=1}{\sum }}{\underline{M}}_{e}$$

where:

(A25) $${\underline{M}}_{e}={\left[\begin{array}{cc} \begin{array}{ccc} \cdots & \cdots & \cdots \\ \cdots & M_{e} & \cdots \\ \cdots & \;\;\cdots & \cdots \end{array} & \begin{array}{ccc} \cdots & \cdots & \cdots \\ \cdots & \cdots & \cdots \\ \cdots & \cdots & \cdots \end{array}\\ \begin{array}{ccc} \cdots \;\; & \cdots & \cdots \\ \cdots \;\; & \cdots & \cdots \\ \cdots \;\; & \cdots & \cdots \end{array} & \begin{array}{ccc} \cdots & \cdots & \cdots \\ \cdots & \cdots & \cdots \\ \cdots & \cdots & \cdots \end{array} \end{array}\right]}_{m\times m}$$

### Appendix A.6. Damping Matrix of the Whole M-Degree-of-Freedom Discrete System

(A26) $$L=\overset{i_{e}}{\underset{e=1}{\sum }}{\underline{L}}_{e}+\overset{i_{k}}{\underset{k=1}{\sum }}{\underline{L}}_{k}$$

where:

(A27) $${\underline{L}}_{e}={\left[\begin{array}{cc} \begin{array}{ccc} \cdots & \cdots & \cdots \\ \cdots & L_{e} & \cdots \\ \cdots & \cdots & \cdots \end{array} & \begin{array}{ccc} \cdots & \cdots & \cdots \\ \cdots & \cdots & \cdots \\ \cdots & \cdots & \cdots \end{array}\\ \begin{array}{ccc} \cdots & \cdots & \cdots \\ \cdots & \cdots & \cdots \\ \cdots & \cdots & \cdots \end{array} & \begin{array}{ccc} \cdots & \cdots & \cdots \\ \cdots & \cdots & \cdots \\ \cdots & \cdots & \cdots \end{array} \end{array}\right]}_{m\times m},$$

(A28) $${\underline{L}}_{k}={\left[\begin{array}{cccccc} \cdots & \cdots & \cdots & \cdots & \cdots & \cdots \\ \cdots & N_{e1k}^{T}\Theta_{e1k}^{T}L_{k}\Theta_{e1k}N_{e1k} & \cdots & \cdots & -N_{e1k}^{T}\Theta_{e1k}^{T}L_{k}\Theta_{e2k}N_{e2k} & \cdots \\ \cdots & \cdots & \cdots & \cdots & \cdots & \cdots \\ \cdots & \cdots & \cdots & \cdots & \cdots & \cdots \\ \cdots & -N_{e2k}^{T}\Theta_{e2k}^{T}L_{k}\Theta_{e1k}N_{e1k} & \cdots & \cdots & N_{e2k}^{T}\Theta_{e2k}^{T}L_{k}\Theta_{e2k}N_{e2k} & \cdots \\ \cdots & \cdots & \cdots & \cdots & \cdots & \cdots \end{array}\right]}_{m\times m},$$

(A29) $$\Theta_{ek}=\left[\begin{array}{cccc} \Theta_{ek}^{*} & 0 & 0 & 0\\ 0 & \Theta_{ek}^{*} & 0 & 0\\ 0 & 0 & \Theta_{ek}^{*} & 0\\ 0 & 0 & 0 & \Theta_{ek}^{*} \end{array}\right],$$

$L_{k}=diag\left(l_{ki}\right),\;i=1,\;\ldots \;,\;6$—matrix of damping coefficients of SDE no. *k*, connecting E-BB no. *e* = *e*1 and E-BB no. *e* = *e*2,
$\Theta_{ek}^{*}={\left[cos\;\alpha_{ekij}\right]}_{3\times 3}$—matrix of direction cosines of angles $\alpha_{ekij}$ between axis $y_{ki}$ of SDE no. *k* and axis $x_{ej}$ of E-BB no. *e*, i=1, …, 3, j=1, …, 3,
$N_{ek}$—matrix of shape functions of E-BB no. *e*, determined for the coordinates of the connection point of SDE no. *k* and E-BB no. *e*.

### Appendix A.7. Stiffness Matrix of the Whole M-Degree-of-Freedom Discrete System

(A30) $$K=\sum_{e=1}^{i_{e}}{\underline{K}}_{e}+\sum_{k=1}^{i_{k}}{\underline{K}}_{k},$$

where:

(A31) $${\underline{K}}_{e}={\left[\begin{array}{cc} \begin{array}{ccc} \cdots & \cdots & \cdots \\ \cdots & K_{e} & \cdots \\ \cdots & \cdots & \cdots \end{array} & \begin{array}{ccc} \cdots & \cdots & \cdots \\ \cdots & \cdots & \cdots \\ \cdots & \cdots & \cdots \end{array}\\ \begin{array}{ccc} \cdots & \cdots & \cdots \\ \cdots & \cdots & \cdots \\ \cdots & \cdots & \cdots \end{array} & \begin{array}{ccc} \cdots & \cdots & \cdots \\ \cdots & \cdots & \cdots \\ \cdots & \cdots & \cdots \end{array} \end{array}\right]}_{m\times m},$$

(A32) $${\underline{K}}_{k}={\left[\begin{array}{cccccc} \cdots & \cdots & \cdots & \cdots & \cdots & \cdots \\ \cdots & N_{e1k}^{T}\Theta_{e1k}^{T}K_{k}\Theta_{e1k}N_{e1k} & \cdots & \cdots & -N_{e1k}^{T}\Theta_{e1k}^{T}K_{k}\Theta_{e2k}N_{e2k} & \cdots \\ \cdots & \cdots & \cdots & \cdots & \cdots & \cdots \\ \cdots & \cdots & \cdots & \cdots & \cdots & \cdots \\ \cdots & -N_{e2k}^{T}\Theta_{e2k}^{T}K_{k}\Theta_{e1k}N_{e1k} & \cdots & \cdots & N_{e2k}^{T}\Theta_{e2k}^{T}K_{k}\Theta_{e2k}N_{e2k} & \cdots \\ \cdots & \cdots & \cdots & \cdots & \cdots & \cdots \end{array}\right]}_{m\times m},$$

$K_{k}=diag\left(k_{ki}\right),\;i=1,\;\ldots \;,\;6$—matrix of stiffness coefficients of SDE no. *k*, connecting E-BB no. *e* = *e*1 and E-BB no. *e* = *e*2.

### Appendix A.8. Time-Dependent Matrix of Transformation from Generalized Coordinates Vector $q_{m\times 1}$ to Cartesian Coordinates y_l__*1*_, y_l__*2*_, y_l__*3*_ of CE No. l

(A33) $$T_{l}\left(t\right)={\left[\begin{array}{ccccc} 0 & \cdots & \Theta_{el}\left(t\right)N_{el}\left(t\right) & \cdots & 0 \end{array}\right]}_{6\times m}\;,$$

where:

(A34) $$\Theta_{el}=\left[\begin{array}{cccc} \Theta_{el}^{*} & 0 & 0 & 0\\ 0 & \Theta_{el}^{*} & 0 & 0\\ 0 & 0 & \Theta_{el}^{*} & 0\\ 0 & 0 & 0 & \Theta_{el}^{*} \end{array}\right]\;,$$

$\Theta_{el}^{*}={\left[cos\;\alpha_{elij}\right]}_{3\times 3}$—matrix of direction cosines of angles $\alpha_{elij}$ between axis $y_{li}$ of CE no. *l* and axis $x_{ej}$ of E-BB no. *e*, i=1, …, 3, j=1, …, 3,
$N_{el}$—matrix of shape functions of E-BB no. *e*, determined for the coordinate of the connection point of CE no. *l* and E-BB no. *e*.

Note: The formulas in Appendix A.1, Appendix A.2 and Appendix A.3 of the Appendix A were developed on the basis of the References [32,33], while the formulas in Appendix A.4, Appendix A.5, Appendix A.6 and Appendix A.7—based on [32].
